# Bibliographical

**Published:** 1868

**Authors:** 


### BIBLIOGRAPHICAL.

---

*Watson Abridged*: *A synopsis of the Lectures on the Principles and Practice of Physic, delivered in King's College, London.* By Thomas Watson, M. D., &c. (Abridged from the last English edition, with valuable additions, &c.) By J. J. Meylor, A. M., M. D. Published by the Author: Philadelphia. 1867.

The above little work of 272 pages has been received from the Author.

As a book of easy reference—having full index of subjects mentioned in it—we are satisfied of its usefulness to the practitioner, who, in the perusal of a few lines, can get the main facts detailed through as many pages of the unabridged work. We well remember the advantages derived from a similar abridgment of Eberle's Practice in the commencement of our professional career, called "Eberle's Notes." We commend Dr. Meylor's book to the medical reader.

---

*Treatment of Diseases of the Throat and Lungs by Inhalations with a new Inhaling Apparatus.* By EMIL SIEGLE, M. D. Translated from the second German edition. By S. NICKLES, M. D. Cincinnati: R. W. CARROLL & Co., Publishers. 1868.

We have for some time been convinced of the great importance of topical treatment for chronic diseases of the respiratory passages, and read with pleasure works on this subject. The above little book will be read with advantage by physicians.

While the complicated apparatus, sometimes used for inhaling the vapor of dilute solutions for such diseases, may be profitably substituted by very simple means for applying to them more concentrated preparations, we hail with satisfaction any improvements in this way.

---

*Signs and Diseases of Pregnancy.* By THOMAS HAWKES TANNER, M. D., F. L. S., &c., &c. From the second and enlarged London edition. Philadelphia: HENRY C. LEA. 1868.

A handsome volume of 482 pages, bearing the above title, has been sent us by the Publishers. The mechanical execution of the work is such as would be expected from this excellent Publishing House; and the subjects indicated on the title page are ably treated of, and in the most fascinating style. Its perusal is more a work of pleasure than of labor. Every medical library should have Tanner's Signs and Diseases of Pregnancy.

*A Manuel of the Dissection of the Human Body.* By LUTHER HOLDEN, F. R. C. S., &c., London. With notes and additions by ERSKINE MASON, M. D., &c. New York: Published by ROBERT M. DEWITT, 13 Frankfort street, New York. 1868.

This work, as the title implies, is a full record of the parts revealed by dissection, and does not treat of any system of orgons in their connection. The nerves, arteries, muscles, &c., of any particular region are given in the order in which the knife reveals them, in which it differs with ordinary works on anatomy, that consider, in connection, all the parts of each general system of the body—such as the nervous, circulatory, &c. This is a convenient book for studying the relations of parts in any particular region.

